# Right ventricular ejection fraction is better reflected by transverse rather than longitudinal wall motion in pulmonary hypertension

**DOI:** 10.1186/1532-429X-12-35

**Published:** 2010-06-04

**Authors:** Taco Kind, Gert-Jan Mauritz, J Tim Marcus, Mariëlle van de Veerdonk, Nico Westerhof, Anton Vonk-Noordegraaf

**Affiliations:** 1 Department of Pulmonary Diseases, VU University Medical Center, Amsterdam, The Netherlands; 2 Department of Physics and Medical Technology, VU University Medical Center, Amsterdam, The Netherlands; 3 Department of Physiology, VU University Medical Center, Amsterdam, The Netherlands

## Abstract

**Background:**

Longitudinal wall motion of the right ventricle (RV), generally quantified as tricuspid annular systolic excursion (TAPSE), has been well studied in pulmonary hypertension (PH). In contrast, transverse wall motion has been examined less. Therefore, the aim of this study was to evaluate regional RV transverse wall motion in PH, and its relation to global RV pump function, quantified as RV ejection fraction (RVEF).

**Methods:**

In 101 PH patients and 29 control subjects cardiovascular magnetic resonance was performed. From four-chamber cine imaging, RV transverse motion was quantified as the change of the septum-free-wall (SF) distance between end-diastole and end-systole at seven levels along an apex-to-base axis. For each level, regional absolute and fractional transverse distance change (SFD and *fractional-*SFD) were computed and related to RVEF. Longitudinal measures, including TAPSE and fractional tricuspid-annulus-apex distance change (*fractional*-TAAD) were evaluated for comparison.

**Results:**

Transverse wall motion was significantly reduced at all levels compared to control subjects (p < 0.001). For all levels, *fractional*-SFD and SFD were related to RVEF, with the strongest relation at mid RV (R^2 ^= 0.70, p < 0.001 and R^2 ^= 0.62, p < 0.001). For TAPSE and *fractional*-TAAD, weaker relations with RVEF were found (R^2 ^= 0.21, p < 0.001 and R^2 ^= 0.27, p < 0.001).

**Conclusions:**

Regional transverse wall movements provide important information of RV function in PH. Compared to longitudinal motion, transverse motion at mid RV reveals a significantly stronger relationship with RVEF and thereby might be a better predictor for RV function.

## Introduction

Pulmonary hypertension (PH) is characterized by increased pulmonary pressure, leading to right ventricular (RV) overload. The course of the illness varies from patient to patient, with the worst prognosis seen in patients with the greatest degree of RV dysfunction [1,2]. This highlights the importance of knowledge of RV function for the determination of prognosis and therapy strategies.

In general, RV ejection fraction (RVEF) is assumed to be a major determinant of systolic RV function, and has been shown to be of prognostic value in PH [3,4]. However, determining RVEF is time consuming and depends on geometric assumptions, and this has limited the application in clinical practice [5,6]. A simpler approach is to approximate RVEF by measuring the tricuspid annular plane systolic excursion (TAPSE). This measure quantifies the longitudinal shortening of the RV and its clinical value has been well established by echocardiography [7-10]. It has also recently been applied using cardiovascular magnetic resonance (CMR) [11].

Less attention has been paid to RV transverse wall motion in the literature [12-14], despite the fact that transverse movements of the RV free wall towards the septum are important in RV ejection (bellows action) [12,15,16].

Therefore, the aim of the present study was to measure RV transverse motion in PH and to assess its relationship with RVEF employing CMR. Longitudinal RV motion measurements were included for comparison purposes. Results in PH patients were compared to control subjects.

## Materials and methods

### Patients and control subjects

The local Ethics Committee of the VU University Medical Center approved the study protocol and all participants gave written informed consent. Between September 2004 and September 2008, 658 patients were referred to our hospital for evaluation of PH. PH was diagnosed according to a standard protocol [17], including right heart catheterization (RHC), and was confirmed when the mean pulmonary artery pressure at rest was >25 mmHg and the pulmonary capillary wedge pressure was < 15 mmHg. Inclusion criteria were: patients with PH with etiologies from WHO group 1 (pulmonary arterial hypertension) or group 4 (chronic thrombotic or embolic disease), and who had undergone CMR within 14 days after RHC had been performed. In total 123 patients met these criteria. Of this group, 15 patients were excluded because some CMR cines were lacking and 7 patients due to technically inadequate images. Thus, in total 101 patients were included in the study. In addition, a total of 29 healthy non-smoking, age and gender matched control subjects were included as a reference group. These healthy subjects had had no RHC.

### Cardiopulmonary exercise testing

In a subset of patients (n = 66), cardiopulmonary exercise testing was performed on an electromagnetically braked cycle ergometer (Rehcor, Lode Groningen, The Netherlands). A progressive increase in pedaling workload (5-20 W/min) was applied until maximum tolerance was reached. Peak oxygen consumption (VO_2,peak_) was considered to express the patient's exercise capacity, which was measured using a metabolic cart (Vmax 229; Viasys, Yorba Linda, CA). To ensure accuracy, the measurements were time-averaged over a minimum of 20 seconds. The equipment was calibrated according the manufacturer's specifications.

### Cardiovascular magnetic resonance

CMR was performed by means of a 1.5T Siemens Avanto MRI system (Siemens Medical Solutions, Germany), equipped with a 6-element phased-array coil. ECG-gated cine imaging was performed using a balanced steady-state free precession pulse sequence, during repeated breath-holds. Long-axis slices were acquired in the four, three and two-chamber views.

The four-chamber image plane was localized by the following steps: a basal short-axis image at end-diastole was used as the first localizer. Orthogonal to this short-axis image, the four-chamber view was obtained by rotating the planning line to such an orientation that it passes through the middle of the mitral and tricuspid valvular rings. The LV vertical long-axis view was used as the second localizer, to assure that the planned four-chamber cine passes through the most apical point of the LV cavity.

Additionally, short-axis slices were obtained with a typical slice thickness of 5 mm and an interslice gap of 5 mm, fully covering both ventricles from base to apex. The MR parameters used were: temporal resolution between 35 to 45 ms, voxel size 1.5 × 1.8 × 5.0 mm^3^, flip angle 60°, receiver bandwidth 930 Hz/pixel, TR/TE 3.2/1.6 ms, matrix 256 × 156.

### Image analysis

**Figure 1:**
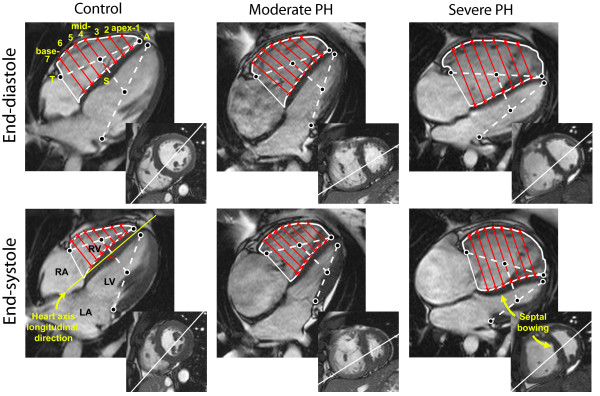
**Four-chamber and short axis views of a healthy subject, a patient with moderate PH and a patient with severe PH**. The figure illustrates how the longitudinal dimension (from tricuspid annulus to apex (TA)) and transverse dimensions (from septum to free-wall (SF)) are determined. Firstly, both in end-diastole and end-systole, the left and right lateral annulus-apex lines were drawn. Secondly, the intersection through the centres of these lines was drawn. SF dimensions were considered parallel to this intersecting line. Thirdly, RV endocardial contours were drawn to determine the SF dimension at seven different levels covering the whole RV (indicated as apex-1 through base-7, with level mid-4 exactly halfway through the RV). The white lines in the short axis views indicate the intersections of the four-chamber views. RV = right ventricle, RA = right atrium, LV = left ventricle, LA = left atrium, A = Apex, T = lateral tricuspid annulus, S = RV endocardial septum, F = RV endocardial free wall.

An apical four-chamber view was used to analyze longitudinal and transverse movements of the RV myocardium (Figure 1). The software for the analysis was implemented in MATLAB release R2008a (The MathWorks, Inc., Natick, United States).

#### Transverse measures

Transverse RV movements were analyzed using a method that quantifies regional changes from septum to free-wall (SF) between end-diastole and end-systole. First, lines were drawn between the right-lateral point of the tricuspid annulus and the apex, and between the left-lateral point of the mitral annulus and the apex. Then, the line intersecting the centers of the left and right lateral annulus-apex lines was drawn (Figure 1), both in end-diastole and in end-systole. SF dimensions were considered parallel to the intersecting line. The advantage of this approach is that clear markers are used to determine the geometric orientation of the SF dimensions. Next, contours were carefully drawn within the compact layer but outside the trabeculated layer of the RV myocardium. If at end-systole the interstices between hypertrophied trabeculae were no longer visible the corresponding boundary line was estimated after careful observation in cine mode.

Subsequently, SF dimensions were computed at seven different levels covering the whole RV cavity, indicated as apex-1 through base-7, with level mid-4 exactly halfway through the RV. For each level, the absolute SF distance change (SFD) was determined, and the fractional SF distance change (*fractional*-SFD) was then computed as SFD divided by the SF dimension at end-diastole.

End-diastole was defined as the onset of the R-wave in the ECG. End-systole was determined visually as the moment of end-shortening of the RV free wall. This definition of end-systole was applied because a time delay often exists between pulmonary valve closure and end-shortening in PH. In this situation leftward septal bowing is observed, where RV end-shortening coincides with maximum bowing[18].

#### Longitudinal measures

Longitudinal RV movements were quantified as modified TAPSE, adapted from echocardiography [11]. CMR TAPSE was computed as the absolute distance change between end-diastole and end-systole of the tricuspid annulus-apex dimension. Fractional tricuspid annulus-apex distance change (*fractional-*TAAD) was calculated as TAPSE divided by the tricuspid annulus-apex dimension at end-diastole.

#### Area change

RV area change in 4-chamber view was quantified from the endocardial contours at end-diastole and end-systole. Fractional area change (FAC) was calculated as the absolute area change divided by the end-diastolic area.

#### RVEF

Endocardial surfaces were carefully manually traced from the stack of short-axis cine images, using Mass Analysis software (MEDIS Medical Imaging Systems, Leiden, The Netherlands) to obtain RV end-diastolic and end-systolic volumes. Trabeculae and papillary muscles were excluded from these measurements. The RV end-systolic image was identified by selecting the smallest ventricular surface. Based on these volumes, RVEF and stroke volume were calculated.

### Statistics

Normal distribution of the data was verified using a normal probability plot and log transformed if necessary. All data are presented as mean ± SD, unless stated otherwise. A p-value < 0.05 was considered statistically significant. Data between controls and patients were compared using the 2-tailed Students t-test for unpaired data or using one-way ANOVA when more than two groups were tested. The Fisher exact test was used for categorical data. Linear regression was performed to test the relationships between the transverse measures (SFD and *fractional-*SFD) and RVEF, and between the longitudinal measures (TAPSE and *fractional-*TAAD) and RVEF. In the regression analysis only patients were included in order to avoid regression bias by the control group.

Receiver operating characteristic (ROC) analysis was used to test sensitivity and specificity of all measures to detect a RVEF less than the median RVEF value in patients. To correct for multiple testing, the threshold for significance was adjusted using Bonferroni correction for families of tests with 0.05 divided by the amount of tests giving the adjusted threshold for significance.

Intra-observer and inter-observer variability of the endocardial wall measurements were assessed using the analysis of agreement method described by Bland and Altman [19]. To this end, the same observer repeated CMR measurements of 10 patients and 10 control subjects within a period of one month to determine the intra-observer variability. A second observer repeated the same measurements to obtain the inter-observer variability.

All statistical analyses were performed with SPSS Statistics 15.0 (SPSS Inc., Chicago, United States).

## Results

### Characteristics of the study population

There was no difference between the 101 PH patients and 29 control subjects with respect to age (PH = 50 ± 15 years vs. control = 46 ± 19 years, p = 0.082) and gender (PH = 68% female vs. control = 67% female, p = 0.35). Table 1 summarizes the clinical and hemodynamic characteristics of the PH patients. Most patients were from WHO group 1 (80%), with the remainder being from group 4 (chronic thromboembolic pulmonary hypertension). Approximately 60% of all patients were medically treated for PH at enrollment, and many of them went through one or more regimens.

**Table 1:** Clinical characteristics and hemodynamics of PH patients.

Clinical characteristic	Value
**Functional status**,	
NYHA functional class II, III, IV (n)	30, 51, 20
6MWD (predicted), m	443 ± 142 (589 ± 102)
Dyspnoe score (Borg index)	4 ± 2
**Diagnosis (n)**	
Idiopathic PAH	41
Familial PAH	9
PAH associated with	
Systemic sclerosis	27
Portal hypertension	1
HIV	2
Chronic thromboembolic pulmonary hypertension	21
**Treatment† (n)**	
Bosentan	17
Bosentan + Sildenafil	15
Epoprostenol	13
Sildenafil	7
Epoprostenol + Sildenafil	7
Sitaxentan	5
Sildenafil + Sitaxentan	4
Treprostinil + Bosentan + Sildenafil	3
Epoprostenol + Sildenafil + Bosentan	3
Treprostinil + Sitaxentan	2
Calcium Antagonist	2
Bosentan + Epoprostenol	2
Treprostinil + Sildenafil	1
**Hemodynamics**	
Heart rate, beats/min	79 ± 13
Mean pulmonary artery pressure, mmHg	46 ± 16
Mean right atrial pressure, mmHg	6 ± 4
Pulmonary capillary wedge pressure, mmHg	8 ± 4
Pulmonary vascular resistance, dyn.s/cm5	632 ± 370
Cardiac output, l/min	5,5 ± 1,9
Cardiac index, l/min/m^2^	3,0 ± 0,9
Mixed venous O_2 _saturation, %	67 ± 9
Cardiopulmonary exercise testing*	
VO_2,peak _(ml/min)	1039 ± 544
VO_2,peak_-predicted (%)	46.0 ± 20.7

*Data obtained in a subset of patients (n = 66). †Note that a considerable number of patients went through different regimens before study enrollment.

NYHA = New York Heart Association functional class; 6MWD = six minute walk distance.

Values are mean ± SD.

Baseline CMR measurements of PH patients and control subjects are presented in Table 2. Values of RVEF were significantly different between the groups, but no significant differences were found for LVEF. However, there was a significant difference for LVEDV between the two groups.

**Table 2:** CMR measurements of control subjects and PH patients.

	Control subjects	PH patients	p-value
CI (l/min/m^2^)	3.3 ± 0.9	2.6 ± 0.9	0.267
HR (bpm)	73 ± 12	82 ± 16	0.107
SV (ml)	72 ± 18	54 ± 21	**0.001**
SV index (ml/m^2^)	35 ± 19	30 ± 11	**0.001**
RVEF (%)	56 ± 8	36 ± 12	**0.001**
LVEF (%)	65 ± 8	66 ± 9	0.689
RVEDV (ml)	126 ± 36	155 ± 61	**0.001**
LVEDV (ml)	119 ± 34	90 ± 29	**0.001**

CI = cardiac index; HR = heart rate; SV = stroke volume; SVI = stroke volume index; RV = right ventricle; LV = left ventricle; EF = ejection fraction; EDV = end-diastolic volume. Values are mean ± SD.

Figure 1 illustrates four-chamber and short-axis views of a healthy subject and two patients with moderate or severe PH.

### Comparison of RV regional wall motion between patients and controls

**Figure 2:**
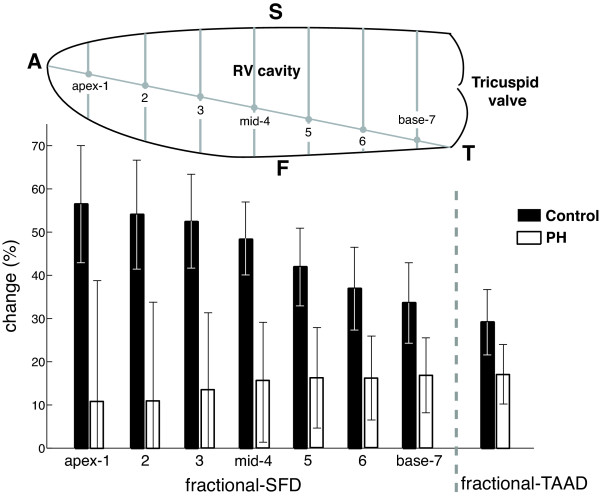
***Fractional*-SFD in control subjects and PH patients, for seven longitudinal levels in the RV, from apex (level 1) to base (level 7)**. *Fractional*-TAAD is shown on the right. In patients, the RV exhibits for each ventricular level approximately the same *fractional*-SFD. In contrast, control subjects show the highest *fractional*-SFD around the apex, with less *fractional*-SFD around the base. For every longitudinal level, differences in fractional change between controls and patients were significant with p < 0.0001. A = Apex, T = lateral annulus of tricuspid valve, S = RV endocardial septum, F = RV endocardial free wall.

Figure 2 shows results of *fractional*-SFD, evaluated for seven levels ranging from apex to base, and *fractional*-TAAD in patients and controls. In PH patients, virtually no regional variation in *fractional*-SFD was found. In contrast, control subjects showed the highest *fractional*-SFD at the apical segment, and the lowest at the basal segment. At all levels, there were significant differences in transverse movements between patients and control subjects (Table 3). From Table 3 it can be seen that for all subjects, SFD values are consistently smaller than TAPSE, but that this does not hold for *fractional*-SFD compared to *fractional*-TAAD due to the shorter end-diastolic transverse dimension (SF) compared to the longitudinal dimension (TAA).

**Table 3:** SF and TA distance at end-diastole and end-systole in control subjects and PH patients

	Control subjects				PH patients			
*Transverse movements*								
level	SF_ed _(mm)	SF_es _(mm)	SFD (mm)	*fractional*-SFD (%)	SF_ed _(mm)	SF_es _(mm)	SFD (mm)	*fractional*-SFD (%)
apex-1	17 ± 5	7 ± 3	10 ± 4	56 ± 14	27 ± 11	25 ± 13	2 ± 7*	11 ± 28*
2	24 ± 5	11 ± 4	13 ± 4	54 ± 13	34 ± 10	31 ± 13	3 ± 7*	11 ± 22*
3	30 ± 5	15 ± 4	17 ± 6	52 ± 10	40 ± 9	35 ± 12	5 ± 6*	13 ± 18*
mid-4	36 ± 5	19 ± 5	19 ± 6	48 ± 9	46 ± 9	40 ± 12	7 ± 6*	16 ± 14*
5	41 ± 5	24 ± 5	18 ± 5	42 ± 9	52 ± 9	44 ± 12	8 ± 6*	16 ± 12*
6	44 ± 6	28 ± 6	16 ± 4	37 ± 10	55 ± 9	46 ± 11	8 ± 5*	16 ± 10*
base-7	45 ± 7	30 ± 7	15 ± 4	33 ± 9	55 ± 9	46 ± 10	9 ± 4*	17 ± 9*
*Longitudinal movements*								
	TAA_ed _(mm)	TAA_es _(mm)	TAPSE (mm)	*fractional*-TAAD (%)	TAA_ed _(mm)	TAA_es _(mm)	TAPSE (mm)	*fractional*-TAAD (%)

-	92 ± 11	65 ± 12	27 ± 7	29 ± 8	99 ± 12	82 ± 14	16 ± 6*	17 ± 7 *
*RV area change*								
	Area_ed _(mm^2^)	Area_es _(mm^2^)	AC (mm^2^)	*fractional*-AC (%)	Area_ed _(mm^2^)	Area_es _(mm^2^)	AC (mm^2^)	*fractional*-AC (%)

-	2840 ± 720	1397 ± 407	1443 ± 374	51 ± 6	3937 ± 1209	2798 ± 1271	1139 ± 421*	31 ± 13*

SF is determined at different levels along an apex to base axis. Values of *fractional*-SFD are also shown in Figure 2. RV area change (AC) and fractional AC are determined in 4-chamber view. Values are mean ± SD.

* Significantly lower compared to control subjects (P < 0.001)

No significant differences were found in longitudinal and transverse motion between the patients from WHO group 1 and 4 (data not shown).

### RV wall motion with reference to RV ejection fraction and exercise capacity

**Figure 3:**
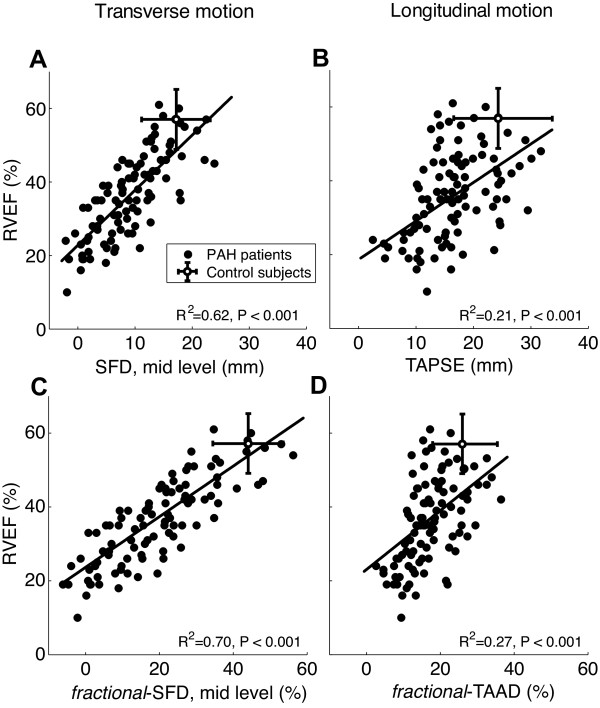
**Regression between RVEF and SFD at mid RV level (A), *fractional-*SFD at mid RV level (C), TAPSE (B), and *fractional*-TAAD (D)**.

*Fractional*-SFD was correlated positively with RVEF at each level of the RV free wall (Table 4), but the strongest correlations were found at level 4 (mid-level; R^2 ^= 0.70, p < 0.001; Figure 3) and level 5 (R^2 ^= 0.66, p < 0.001). Weaker relationships were found for SFD, *fractional*-TAAD and TAPSE (Figure 3 and Table 4).

**Table 4:** Linear regression of transverse and longitudinal parameters, and RVEF in PH patients.

	level	R^2^
*Transverse movements*		

*fractional*-SFD	apex-1	0.30*
	2	0.39*
	3	0.55*
	mid-4	0.70*
	5	0.66*
	6	0.51*
	base-7	0.32*
SFD	apex-1	0.30*
	2	0.35*
	3	0.48*
	mid-4	0.62*
	5	0.55*
	6	0.37*
	base-7	0.17*
*Longitudinal movements*		

*fractional*-TAAD	-	0.27*
TAPSE	-	0.21*
*RV area change*		

AC	-	0.25*
*fractional*-AC	-	0.76*

Data are determined in PH patients only. Values are mean. RV area change (AC) and fractional AC are determined in 4-chamber view.

* Significantly lower compared to control subjects **(P < 0.001).**

**Figure 4:**
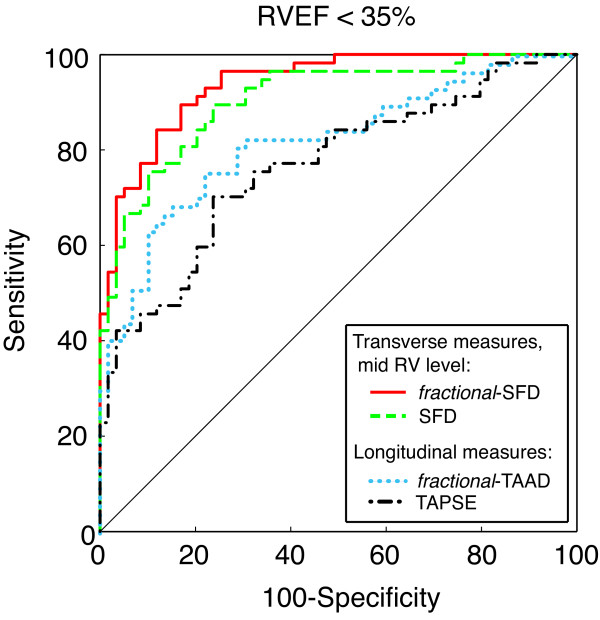
**Receiver operating characteristic curves show ability of *fractional*-SFD at mid RV, SFD at mid RV, *fractional*-TAAD and TAPSE to detect a RVEF below the median value (< 35%)**.

Figure 4 and Table 5 show the results of ROC-analysis, indicating that *fractional*-SFD at mid RV is a sensitive and specific indicator of depressed RVEF below the median value in patients (< 35%, range 10-61%, range control subjects: 41-77%). Compared to *fractional*-SFD, there were statistically significant differences among the areas under the curves for SFD (p = 0.005), *fractional*-TAAD (p = 0.003) and TAPSE (p < 0.001) after Bonferroni correction.

**Table 5:** Diagnostic performance of transverse and longitudinal parameters for detection of depressed RVEF below the median value (< 35%).

	AUC*	Standard error	Cutoff value	Sensitivity† (%)	Specificity† (%)	P-value†
*Transverse measures*						
fractional SF level mid-4	0.938 [0.892, 0.985]	0.0236	17.7%	84.2	88.1	< 0.001
ΔSF SF -mid 4	0.906 [0.849, 0.963]	0.0291	7.4 mm	80.7	83.1	< 0.001
*Longitudinal measures*						

fractional TA	0.819 [0.741, 0.896]	0.0397	17.2%	75.4	78.0	< 0.001
TAPSE	0.767 [0.680, 0.854]	0.0442	16.3 mm	70.2	76.3	< 0.001

*Data is shown with the 95% confidence interval within the brackets.

†At receiver operating characteristic curve analysis.

**Figure 5:**
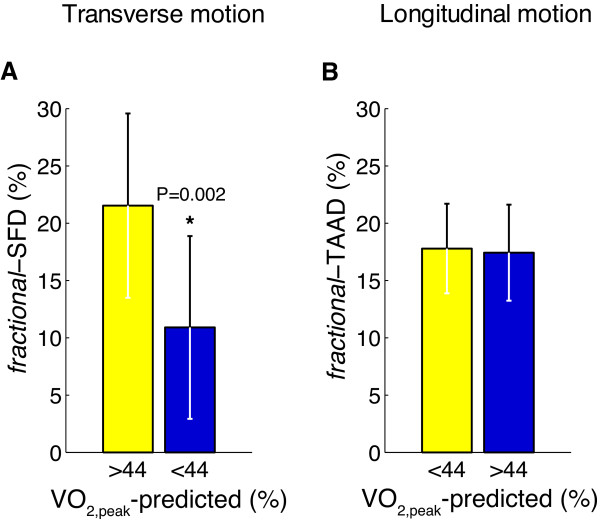
**Bar graphs to compare values of VO_2,peak_-predicted above and below the median value (44%) in comparison with *fractional*-SFD (A) and *Fractional*-TAAD (B)**. VO_2,peak_-predicted values were obtained in a subset of patients (n = 66).

In a subset of patients (n = 66) exercise capacity was measured using VO_2,peak _and VO_2,peak_-predicted (Table 1). Figure 5 illustrates VO_2,peak_-predicted below and above the median value (44%, range: 10-110%) in comparison with *fractional*-SFD and *fractional*-TAAD. A significant difference was found for *fractional*-SFD (p = 0.002) but not for *fractional*-TAAD.

### Variability

**Figure 6:**
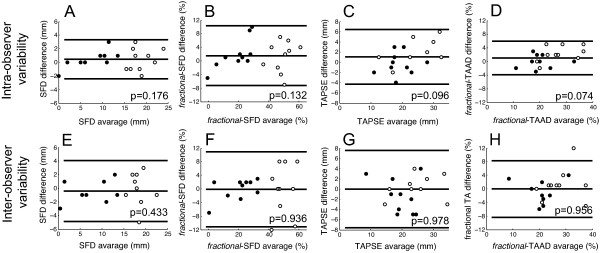
**Analysis of agreement with Bland-Altman plots to illustrate the intra- and inter-observer variability of SF (A and E), fractional SFD (B and F), TAPSE (C and G), and *fractional*-TAAD (D and H)**. Mean difference and 95% limits of agreement are shown. Solid dots: PH patients; empty dots: control subjects.

Figure 6 shows the intra- and inter-observer variability using Bland-Altman plots. The intra- and inter-observer variability for all longitudinal and transverse measures was not statistically significant.

## Discussion

We have evaluated regional transverse motion of the RV myocardium, defined as movements of the RV free-wall to the septum, in a group of PH patients and control subjects. The most important finding of this study was that RVEF was more closely related to fractional transverse movements than to longitudinal movements. The former might consequently be used as an alternative measure of RV pump function. In addition, we observed that fractional transverse movements in control subjects were largest near the apex, but smaller near the base of the heart, while PH patients showed consistently smaller movements and little regional variation.

### RV ejection fraction

A significant relationship between *fractional*-SFD and RVEF was found for all levels, with the strongest relation at mid RV level (R^2 ^= 0.70, p < 0.001). The relationship between TAPSE and RVEF, however, was much lower (R^2 ^= 0.21, p < 0.001). Although this value corresponds to what was found by Kjaergaard et al. [20] (R^2 ^= 0.23), other studies have reported higher correlations (R^2 ^> 0.38) [10,11]. This difference might be explained by the inclusion of control subjects in their regression analysis.

From a practical perspective, the measurement of *fractional*-SFD appears clinical useful. A cutoff value of 17.7% was used to differentiate between patients with a low (< 35%) or preserved (>35%) RVEF (sensitivity of 84.2%; specificity of 88.1%), which had a significantly higher discriminatory power compared to SFD, *fractional*-TAAD or TAPSE (Figure 4; Table 5). Because RVEF is of prognostic importance [3,4] and our results showed that *fractional*-SFD has a high predictive value for RVEF, *fractional*-SFD might be useful in clinical practice.

### Functional considerations

The importance of transverse wall movements in ejection was first acknowledged by Rushmer et al. [15]. They described that although longitudinal movements are easily identified, they are probably much less important for ejection than compression of the RV chamber by movement of the free wall toward the septum (bellows action). However, only a few studies have been performed on (regional) RV transverse wall motion and its contribution to RV function [12-14,21,22] and none of these were performed in PH. Moreover, while some reports examined transverse movements just below the tricuspid valve [21,22], our results, in PH, showed that loss of transverse motion at this level was lower compared to the other regions (Figure 2; Table 3). Evidence for a hypokinetic apex in PH was shown earlier by strain analysis employed by tissue Doppler echocardiography [23-25]. However, this has not been observed using MR myocardial tagging [26].

Considering the transverse measures at mid RV a remarkably large difference exists between PH patients and control subjects (figure 2 and table 3). Moreover, this difference is much larger than is found for the longitudinal measures. To be able to explain these results accurately, the definitions of the measures need to be considered in more detail.

SFD is defined as the difference between end-diastolic and end-systolic SF dimension, and thereby quantifies disturbances of RV free-wall movements. Additionally, paradoxical leftward septal bowing increases the end-systolic SF dimensions and, as a consequence, also affects SFD. Thus, especially in severe patients the existence of a septal bowing may contribute to the large differences seen between patients and controls.

*Fractional*-SFD is defined as SFD divided by the end-diastolic SF dimension. The unusually high end-systolic pressure in the lumen of the RV in patients with PH affects the cross-sectional shape of the cavity. Whereas it normally tends to be crescent shaped in cross section, it adopts a more circular cross section when contracting against pressure that approaches or exceeds that of the left ventricle (Figure 1). This alteration of geometry mainly affects transverse rather than longitudinal dimensions, and therefore contributes more to reduction of *fractional*-SFD rather than *fractional*-TAAD. In addition to any impairment of RV myocardial contractility, this "transverse" dilation may contribute to the large difference between PH patients and controls.

### Anatomical considerations

Despite several anatomical studies, the relationship between fiber orientation and right-heart mechanics is still not completely clear [27,28]. Presently, there is increasing consensus that both the septum and RV free wall play an important role in RV contraction. The septum consists of oblique longitudinal fibers with spiral architecture [29], resulting in the twisting motion required for efficient ejection against increased vascular resistance. In contrast, the predominant transverse fiber orientation of the RV free wall leads to circumferential compression or bellows action, which maintains RVEF with normal pulmonary artery pressure [28,30,31].

In the setting of pulmonary vascular disease, it has been shown that the fiber orientation of the septum becomes more transverse [30]. An altered fiber orientation in the RV free wall is also likely to occur as the RV dilates. This dilation is profound toward the apical segments and results in an increased apical angle (Figure 1) [32]. Changes in fiber direction in the free wall are supported by a study of Pettersen et al. [33] in which MR strain analysis was applied to patients with a systemic RV. There results indicated a predominant circumferential over longitudinal free wall shortening at mid RV, while the reverse has been observed in healthy control subjects [33,34].

Therefore, we hypothesize that disturbed RV wall motion in PH can be explained by impaired RV myocardial contractility due to altered fiber orientation. However, further studies are needed to explain how changes of fiber geometry contribute to both longitudinal and transverse wall motion.

### Practical implications

RV function is the primary determinant of prognosis in PH. Therefore, clinicians need simple and reproducible tests to assess RV function in order to improve their management of PH. In general, RVEF is assumed to be a major determinant of systolic RV function. However, its determination is time consuming and is limited by large inter and intra-observer variability. TAPSE has been shown to correlate with RVEF and has been considered a simple method for semi-quantitative assessment of RV function. In this study we have showed that *fractional*-SFD around mid RV level is more strongly correlated with RVEF. Since SF dimension around mid level is as simple to measure as TAPSE (only two points are needed), it is an easy and accurate way to assess RV function with CMR. The clinical value of the transverse measures would be even stronger if these could be measured using echocardiography. This should be investigated in future research.

### Limitations

Some limitations of the current study should be noted. Firstly, geometry and heavily trabeculated myocardium of the RV make it sensitive to errors in the determination of endocardial definition. Secondly, four-chamber views were acquired with equal geometric orientation at end-diastole and end-systole, without accounting for through plane motion. It is unknown whether RV torsion may affect the end-systolic dimension. A previous study reported regional differences in rotation in the systemic RV. However, when values were averaged over different segments, this resulted in almost absent rotation at the basal and the apical level with no absolute global ventricular torsion [35].

## Conclusions

In PH, transverse myocardial motion is significantly declined. Moreover, measures of transverse movement at mid RV reveal a significant relationship with RVEF, which is much stronger than for measures of longitudinal movement. Since *fractional*-SFD at mid level is as feasible and reliable to measure as TAPSE, it might be a better predictor of RV function in PH. Further studies are needed to evaluate the usefulness in clinical practice.

## Abbreviations

CMR: Cardiovascular magnetic resonance; LV: left ventricle; PH: pulmonary hypertension; RV: right ventricle; RVEF: right ventricle ejection fraction; SF: septum-free wall dimension; TAA: tricuspid annulus-apex dimension; SFD: septum to free wall distance change; TAAD: tricuspid annulus to apex distance change; TAPSE: tricuspid annular systolic excursion.

## Competing interests

The authors declare that they have no competing interests.

## Authors' contributions

TK: study design, manuscript preparation and revision, writing the Matlab software, data collection, analysis and interpretation. GJM: study design, data collection and interpretation. MV: data collection, analysis and interpretation, manuscript revision. JTM: data collection, manuscript revision. AVN and NW: study design, data interpretation, manuscript revision. All authors read and approved the final manuscript.
